# Are Soy Products Effective in DMD?

**DOI:** 10.1371/currents.md.0439d464ca3344340ac9a7182a6ea28a

**Published:** 2018-03-27

**Authors:** Steve J Winder, Gemma Marston

**Affiliations:** Department of Biomedical Science, University of Sheffield, Sheffield, United Kingdom; School of Health Sciences, Liverpool Hope University, Liverpool, United Kingdom

## Abstract

**INTRODUCTION:**

In addition to their nutritional value, processed soy bean extracts contain several activities with potential therapeutic benefits. These include anti-oxidants, and tyrosine kinase and protease inhibitory activity. There are also anecdotal reports of health benefits of soy products in alleviating DMD symptoms.

**METHODS:**

Mdx mice were fed a control soy-free diet or the same diet containing either a proprietary soy preparation (Haelan 951), purified soy isoflavones, purified Bowman-Birk protease inhibitor or a combination of isoflavones and Bowman-Birk inhibitor. Mice were tested for their wire hanging ability at the start of the diet regimen and every 4 weeks until week 12 of treatment.

**RESULTS AND DISCUSSION:**

The diet containing Bowman-Birk inhibitor was the only one to show a significant and sustained improvement over the 12 weeks of the study. All other dietary additions; Haelan 951, isoflavones and isoflavones with Bowman-Birk inhibitor, were not significantly different from each other or from control. The effectiveness of Bowman-Birk inhibitor in mdx mice clearly warrants further study.

## Introduction

The health benefits of soy products have been recognised for centuries, with anecdotal and scientific evidence of beneficial effects in a wide range of disorders and conditions from diabetes and cancer to heart disease and inflammation^1^. Unprocessed soy beans contain high levels of phytic acid, an anti-nutrient that sequesters vitamins and minerals, and perturbs inositol lipid metabolism, which make it unsuitable for human consumption in large quantities. Soy is therefore most often consumed in fermented form, such as the familiar soy sauce, miso and tempeh or by processing a protein and fat extract of the beans, as in tofu or textured soy protein.

Nutraceutically soy is suggested to have various properties, but the precise chemical basis of many of these activities is poorly defined. The most recognised activities are those of the Bowman Birk inhibitor (BBI) and the isoflavones such as genistein. BBI is an 8 kDa peptide which is a non-competitive dual inhibitor of trypsin and chymotrypsin, it is acid and heat stable and can not only survive passage through the stomach but also passes the gut wall intact and can therefore have systemic effects^2^. BBI is capable of inhibiting many different cellular proteases, though it is thought that its main cellular activity is through inhibition of the chymotrypsin like activity of the proteasome^3^. Genistein, daidzein and glycitein are the principal isoflavones of soy, with well recognised and scientifically tested antioxidant, phytoestrogen and kinase inhibitory properties^4^^, ^5 Genistein is available cheaply and taken widely as a health supplement.

Amongst the DMD community there is significant use of a fermented soy product, Haelan 951, a commercially available health supplement http://www.haelan951.com/. In addition to its nutritional value – high in amino acids, essential fatty acids and vitamins, and low phytic acid content, it contains significant amounts of all 3 isoflavones and BBI. The benefits of Haelan 951 in DMD are entirely anecdotal, with no scientific study being conducted into its use, with only 2 published papers concerning its potential benefits. A case report of a single cancer patient^6^, and a study which suggested some effect in reducing the growth of pancreatic cancer cells in vitro^7^.

On the other hand, there is an abundance of published literature investigating the benefits of genistein 4, and BBI^8^^, ^9 usually given in the form of a Bowman Birk Inhibitor concentrate (BBIC), though only a handful with direct relevance to DMD. In one study in mdx mice BBIC was revealed to provide some benefit to mdx pathophysiology^10^ including benefits to muscle function. While investigation into the antioxidant properties of purified genistein did reveal some modest effects on muscle pathology and biochemical status in mdx mice, the mechanism of action was not elucidated precisely^11^^, ^12.

## Methods

All mdx mice were fed standard mouse chow from weaning. At 12 weeks of age male mdx mice were assigned randomly to treatment groups (n=8 per treatment) and fed the test diets ad lib for a further 12 weeks. Diets comprised (i) the control AIN-93G diet alone or AIN-93G containing (ii) Haelan 951 (25mg/g; http://www.haelanhealth.com), or (iii) Bowman Birk Inhibitor (0.75mg/g; 95% pure T9777 Sigma Chemical Company), or (iv) the isoflavones genistein, daidzein and glycitein in the ratio 23:9:3 (17.5µg/g; LC Labs) or (v) a combination of BBI and the isoflavones. The amount of diet eaten and the mice were weighed every week from commencement of treatment and mice were subjected to wire hang test^13^ at 0, 4, 8 and 12 weeks of treatment.

## Ethics Statement

Mdx mice were housed in the University of Sheffield animal facility according to national and international best practice guidelines for research using animals. All procedures were approved by the University of Sheffield Animal Welfare Committee and carried out under UK Home Office Project License (PPL 60/4453, Animals in Scientific Procedures Act, 1986).

## Results and Discussion

There were no significant differences in diet consumption or in mouse weight gain between the control and experimental diets over the course of the 12 weeks of treatment, nor were there any observable adverse effects on the mice. Comparison of wire hang times over the 12 weeks of treatment using the maximum holding impulse (weight x time)^13^ relative to the start of treatment (100%) as a measure revealed a slight decline in wire hang times in animals on the control diet (Figure 1). Isoflavones and isoflavones with BBI showed a slight and sustained increase in wire hang times over the course of the treatment, but this was not significant from control, whereas Haelan 951was essentially the same as control except at 8 weeks where was a slight but non-significant increase (Figure 1). BBI alone however showed a sustained and significant increase in wire hang times at 4, 8 and 12 weeks of treatment reaching a peak at 8 weeks, followed by decline at 12 weeks (Figure 1). The reason for the decline is not clear from the wire hanging data alone. Given that the dose of BBI administered was based on a calculation of the theoretical amount of actual BBI administered in previous studies using BBIC^10^ the amount of pure BBI and its relative activity and absorption in its pure form, may have resulted in a different pharmacokinetic profile in the tissue leading to some sort of cytotoxic effect at this dose at longer time points. In order to investigate this a follow on dose ranging study would be required, along with pharmacokinetic analysis of the amounts of BBI actually present in the serum and tissues of the treated mice. Comparing the effect of the 4 diets to control at the 12 week treatment time point (Figure 2), demonstrates the spread of the data within in each treatment group but again shows the significant improvement obtained with the diet containing BBI only, whereas Halean 951, the isoflavones and isoflavones with BBI showed no significant difference as compared to control.

**Longitudinal analysis of wire hanging ability in groups of 6-8 mice fed the indicated diets.**
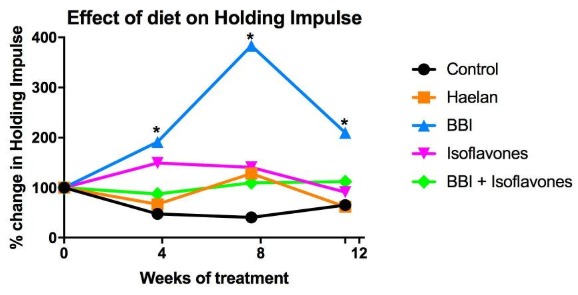
The diets tested were: (i) control AIN 93G diet alone, or 4 test diets of AIN 93G containing (ii) Haelan 951 (Haelan; 25mg/g), or (iii) Bowman Birk Inhibitor (BBI; 0.75mg/g), or (iv) isoflavones (17.5µg/g), or (v) a combination of BBI and the isoflavones (BBI+Isoflavones). Data plotted are mean from groups of 7-8 mice for the % change in maximum holding impulse (HI) for each individual mouse (longest wire hang in seconds/mouse mass in grams) taking the holding impulse at day 0 of treatment as 100%. One way repeated measures ANOVA were performed using GraphPad Prism software. * p<0.05, error bars have been omitted for clarity. Full data are available in the Data Availability Statement.

**Box whisper plot representation of the data in Figure 1 at week 12 of treatment.**
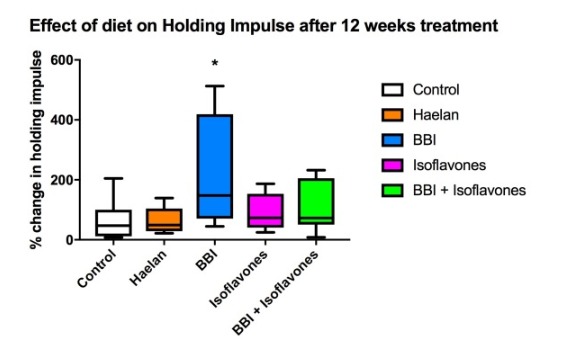
Box whisper plot representation of the data in Figure 1 at week 12 of treatment. All other parameters as for Figure 1. One way ANOVA and Kruskal Wallis test were performed using GraphPad Prism software. * p<0.05.

A previous study examining the effects of 3 months of feeding BBIC on mdx mouse muscle pathophysiology demonstrated a significant improvement in several parameters of muscle function^10^. There was an approximately 25% and statistically significant improvement in EDL muscle mass, cross sectional area, tetanic force and force drop on repeated eccentric contraction. Total mouse mass was 10% higher (but not significant), relative EDL muscle mass was significantly higher, and whilst twitch force was also 25% higher the difference was not significant. There was no change in specific tension. Our findings therefore that purified BBI fed to mice at an equivalent dose to that administered in the Morris study^10^ resulted in a significant increase in wire hang times, is in line with their findings. However what is more difficult to rationalise is why the combination of BBI and isoflavones did not produce a significant improvement in wire hang times.

Both BBIC and Halean 951 contain significant amounts of BBI and isoflavones. Depending on the preparation method used, BBIC contains approximately 110mg/g BBI and 4.54 mg/g isoflavones^14^. Haelan 951 contains a 6-fold lower amount of isoflavones (0.69 mg/g) but in very similar ratios to BBIC. Genistin, diadzin and glycitin and their metabolites are present at 1 : 0.57 : 0.16 in BBIC^14^ and at 1 : 0.41 : 0.14 in Haelan 951 (http://www.haelanhealth.com/haelan-951-fermented-soy/), there is no measure of the amount of BBI in Haelan 951, though the manufacturers’ claim a ‘high rate of the Bowman Birk protease inhibitor compound’ (http://www.haelanhealth.com/haelan-951-test-results/). Given that our rationale was to compare Haelan 951 with purified forms of its potential active ingredients, namely BBI and isoflavones, we used a dose of isoflavones in this study equivalent to the amount in Haelan 951.

A previous study investigating the effects of the predominant soy isoflavone genistein in mdx, used doses up to 2 mg/kg IP daily^11^, this is approximately twice the calculated daily dietary intake of total isoflavones (875 µg) of the mice used in this study. In the study by Messina and colleagues, they reported a siginificant increase in grip strength, and improvements in various parameters of muscle pathology including reduced serum creatine-kinase levels, markers of oxidative stress and muscle necrosis and enhanced regeneration, suggesting that genistein alone could be beneficial in mdx. We did not see any beneficial effect of combined isoflavones in our study, but then at a lower dosing regimen, 2 mg/kg IP, 3 times per week compared to 2 mg/kg IP daily, Messina and colleagues also saw no significant improvement^11^. This would suggest that the dose of isoflavones alone required to achieve a beneficial effect is critical, and/or that isoflavones other than genistein, such as daidzin and glycitin might have deleterious effects, either when used alone or in combination with BBI.

## Conclusion

From the data obtained in this study, we saw no beneficial effect of Haelan 951 or isoflavones, and only Bowman-Birk Inhibitor alone produced any significant improvement in muscle function. Further studies into the dose-activity relationship of BBI and isoflavones in mdx mice are therefore clearly warranted.

## Competing Interests Statement

G Marston declares that no competing interests exist. SJ Winder is a member of the Editorial Board of PLOS Currents Muscular Dystrophy. However he he took no part in the peer review or editorial decision making with respect to this manuscript.

## Data Availability Statement

Metadata for the study can be found at https://doi.org/10.15131/shef.data.5947858

## Corresponding Author

Steve J Winder is the Corresponding Author. They can be reached via the following email address: s.winder@sheffield.ac.uk
